# Novel plasma pTau205 and BD‐tau prototype assays on LUMIPULSE^®^ G1200

**DOI:** 10.1002/alz.095460

**Published:** 2025-01-09

**Authors:** Jeroen Vanbrabant, Maxime Van Loo, Charlotte Lambrechts, Sherif Bayoumy, Daniel Antwi‐Berko, Wiesje M. van der Flier, Inge M.W. Verberk, Charlotte Teunissen, Erik Stoops

**Affiliations:** ^1^ ADx NeuroSciences NV, Ghent Belgium; ^2^ Neurochemistry Laboratory, Department of Laboratory Medicine, Amsterdam UMC, Vrije Universiteit Amsterdam, Amsterdam Neuroscience, Amsterdam Netherlands; ^3^ Alzheimer Center, Department of Neurology, Amsterdam UMC, Vrije Universiteit Amsterdam, Amsterdam Neuroscience, Amsterdam Netherlands

## Abstract

**Background:**

Among the different phospho‐tau sites, phosphorylation at Threonine 205 (pTau205) in CSF has been shown to be closely associated with Tau‐PET (Lantero‐Rodriguez et al. 2024; Barthélemy et al. 2023). Plasma brain‐derived tau (BD‐tau) has been shown to be an improved marker for amyloid‐associated neurodegeneration (Gonzalez‐Ortiz et al., 2024) compared to total tau since this reflects tau from CNS origin only. We aimed to develop prototype LUMIPULSE G assays for quantification of BD‐tau and pTau205 in EDTA plasma, to ultimately determine different biological stages of Alzheimer’s disease (AD).

**Method:**

Prototype assays were developed using commercial mAbs to capture pTau205 and BD‐tau, respectively, combined with an alkaline phosphatase‐conjugated Fab fragment digested from ADx N‐terminal recombinant mAb RD‐073. Assay intra‐ and inter‐run precision, and freeze‐thaw (F/T) stability were determined. Assays were tested in an exploratory cohort using EDTA plasma samples from 50 AD patients (defined by CSF biomarker profile) and 50 healthy donors from the Dutch Brain Research Registry (Hersenonderzoek.nl).

**Result:**

Levels of pTau205 and BD‐tau were within the measuring range in both control [pTau205: 0.019‐0.087 pg/mL; BD‐tau: 1.8‐6.5 pg/mL] and AD cohorts [pTau205: 0.029‐0.31 pg/mL; BD‐tau: 2.2‐20.7 pg/mL] with 100 µL undiluted and 8‐fold diluted EDTA plasma. Both assays demonstrated robust inter‐ and intra‐run precision below 15% CV. No change in concentration was observed for both analytes for up to 5 F/T cycles. The assays demonstrated moderate clinical performance in this cohort with an AUC of 0.65 [95% CI 0.54‐0.76] for pTau205 and 0.70 [95% CI 0.60 – 0.80] for BD‐tau. Both assays correlated significantly with pTau181 measured with a Quanterix Homebrew Simoa assay [pTau205: Spearman r 0.46; p<0.0001; BD‐tau: Spearman r 063; p<0.0001].

**Conclusion:**

Prototype LUMIPULSE G plasma assays for pTau205 and BD‐tau were developed using specific capture mAbs combined with an N‐terminal pan‐tau detector. The assays could measure both biomarkers in all samples from a clinical plasma cohort comprising 50 controls and 50 CSF‐biomarker confirmed AD patients. Further exploration in larger cohorts, including those characterized by Tau‐PET and Braak staging, is warranted to see whether these plasma markers provide extra information over the AD stages.

